# Corresponding Assessment Scenarios in Laboratory and on-Court Tests: Centrality Measurements by Complex Networks Analysis in Young Basketball Players

**DOI:** 10.1038/s41598-020-65420-3

**Published:** 2020-05-25

**Authors:** C. A. Gobatto, R. S. Torres, F. A. Moura, S. A. Cunha, C. B. Giometti, G. G. Araujo, F. A. B. Sousa, F. B. Manchado-Gobatto

**Affiliations:** 1 Laboratory of Applied Sport Physiology, School of Applied Sciences, University of Campinas, Limeira, SP Brazil; 2 Department of ICT and Natural Sciences, Norwegian University of Science and Technology, Alesund, Norway; 3 Department of Sport Sciences, State University of Londrina, Londrina, PR Brazil; 4 Department of Sport Sciences, University of Campinas, Campinas, SP Brazil; 5 Institute of Physical Education and Sport, Federal University of Alagoas, Maceio, AL Brazil

**Keywords:** Computational biology and bioinformatics, Physiology

## Abstract

Besides technical and tactical aspects, basketball matches involve high aerobic and anaerobic capacities, conferring the final performance of a team. Thus, the evaluation of physical and technical responses is an effective way to predict the performance of athletes. Field and laboratory tests have been used in sports. The first involving high ecological validity and low cost, and the second, greater control and accuracy but not easy application, considering the different preparation phases in a season. This study aimed, through complex networks analysis, to verify whether centrality parameters analysed from significant correlations behave similarly in distinct scenarios (laboratory and on-court), emphasizing aerobic and anaerobic physical parameters and technical performances. The results showed that, in a compelling  analysis involving basketball athletes, the studied centralities (degree, betweenness, eigenvector and pagerank) revealed similar responses in both scenarios, which is widely attractive considering the greater financial economy and lower time when applying tests in the field.

## Introduction

In basketball games, despite the large participation of the aerobic energy system during matches^1,2^, undoubtedly the high power tasks, which include fast running and vertical jumps, predominate the actions and define the final results in competitions^3^. Thus, in terms of physical capacities to be evaluated in athletes of this modality, remarkable emphasis has been given to the more anaerobic parameters, which include the use of anaerobic power determination protocols^3^. Still, in basketball matches and in addition to this physiological aspect, high demand for tasks includes accelerations, decelerations and changes in direction in the more than 1000 intermittent actions (one every 2 seconds) performed during the game^4^. Together, these aspects are even more critical in young athletes, as they need, through physical and technical training, to develop physical conditions capable of supporting such demands in high performance and preventing injuries^5^. In this sense, the employment of more appropriate ergometers on data acquisition related to running force, velocity and power should better guide the training and mechanical and metabolic evaluation processes specific to anaerobic power. The semi-tethered^6,7^ and tethered^8–10^ running performed in the field and laboratory includes a high-frequency signal acquisition system with elevated sensitivity and accuracy, allowing, especially at the high-level performance, to obtain trustworthy and detailed information in an inter- and intra-athlete evaluation process. On the other hand, although such evaluations performed, especially inside laboratories, can be considered relevant to the development of performance sport and used in different parts of the world, it is necessary that other and complementary models of evaluation and implementation of training loads be applied. These field protocols that can be present in the routine training sessions include slightly less robust evaluations, but which define greater methodological simplicity and ecological validity in their accomplishments. In the laboratory, the maximal 30 s tethered running test was developed to give greater specificity to this movement, in an analysis pattern similar to that proposed by Bar-Or^11^ in the cycle ergometer, and has been applied with very good resolutions^12,13^. In a very well applied field scenario, the running anaerobic sprint test (RAST) was suggested to obtain the same parameters as those of the laboratory, allowing greater agility in the anaerobic parameter evaluation processes. The RAST was validated^14^ and applied to basketball in its original form^15^ and adapted to the dimensions of the basketball court^16^, obtaining success in the evaluation of athletes of this modality. However, it is doubtful for both laboratory and basketball court scenarios whether such assessments are indeed compatible and appropriate to the sport when included in a more dynamic model that may involve aerobic capacity as well as anthropometric and performance parameters, such as the vertical jump, and determining the technical skill of shooting the basketball. This open question was addressed in the present study by applying centrality distributions in complex networks. Thus, the objective of the present study was to apply and compare, in two different scenarios L (laboratory, all-out 30 s, AO30s) and C (on-court, RAST), the degree, betweenness, eigenvector and pagerank centrality metrics in a complex networks model capable to verify the relationship of mechanical anaerobic responses obtained in each scenario (L and C) with the other (common) physiological, anthropometric and technical parameters in young male basketball athletes. It was hypothesized that the centrality measurements showed similar responses between both the L and C scenarios.

## Methods

Thirteen male basketball players participated in the study (age 15 to 20 years old,  body mass 71.7 ± 11.3 kg,  height 1.8 ± 0.1 m,  fatty mass 8.4 ± 5.0%). All subjects have participated in the sport for at least 2 years, training daily (5 days a week) and participating uninterruptedly in regional- or state-level competitions in the last 12 months. The subjects were basketball players of the base teams of two traditional Brazilian high-level clubs of this sport. The basketball players did not present pathologies or aggravations, as well as recent osteoarticular injuries. The subjects were recruited by invitation and signed or had their legal guardian sign their agreement to participate in the study. This study was conducted in agreement with ethical recommendations of the Declaration of Helsinki, and all experiments were approved by the Research Ethics Committee of the School of Medical Sciences (protocol number 69680217.3.0000.5404).

### Experimental design

For this study, three evaluation sessions were accomplished. Participants were instructed to replicate their food intake prior to each experimental trial. The participants were asked to refrain from exhaustive exercise as well as ingestion of alcohol and neuroexcitatory substances 48 h prior to each evaluation session. These recommendations were taken to avoid or at least minimize bias in the results. During the first session, the participants underwent anthropometric and body composition evaluations. Body density was estimated using the generalized equation of Jackson and Pollock^17^ (height, body mass and abdominal, suprailiac, tricipital and subscapular skinfolds) and converted to body fat percentage using the equation of Siri^18^. Then, the athletes underwent a standardized warm-up consisting of low intensity running on a treadmill (5 minutes at 7 km/h) followed by three countermovement jumps on a force platform. After this, the athletes were submitted to a tethered running familiarization on a flat non-motorized treadmill (NMT). This familiarization consisted of three maximal 10 s running with two minutes rest among efforts.

At the second visit to the laboratory, the participants were kept at rest for 10 minutes, and then underwent the same warm-up as the first visit. After a 5 minutes recovery, the athletes were submitted to all-out running for 30 s on the NMT.

**Figure 1:**
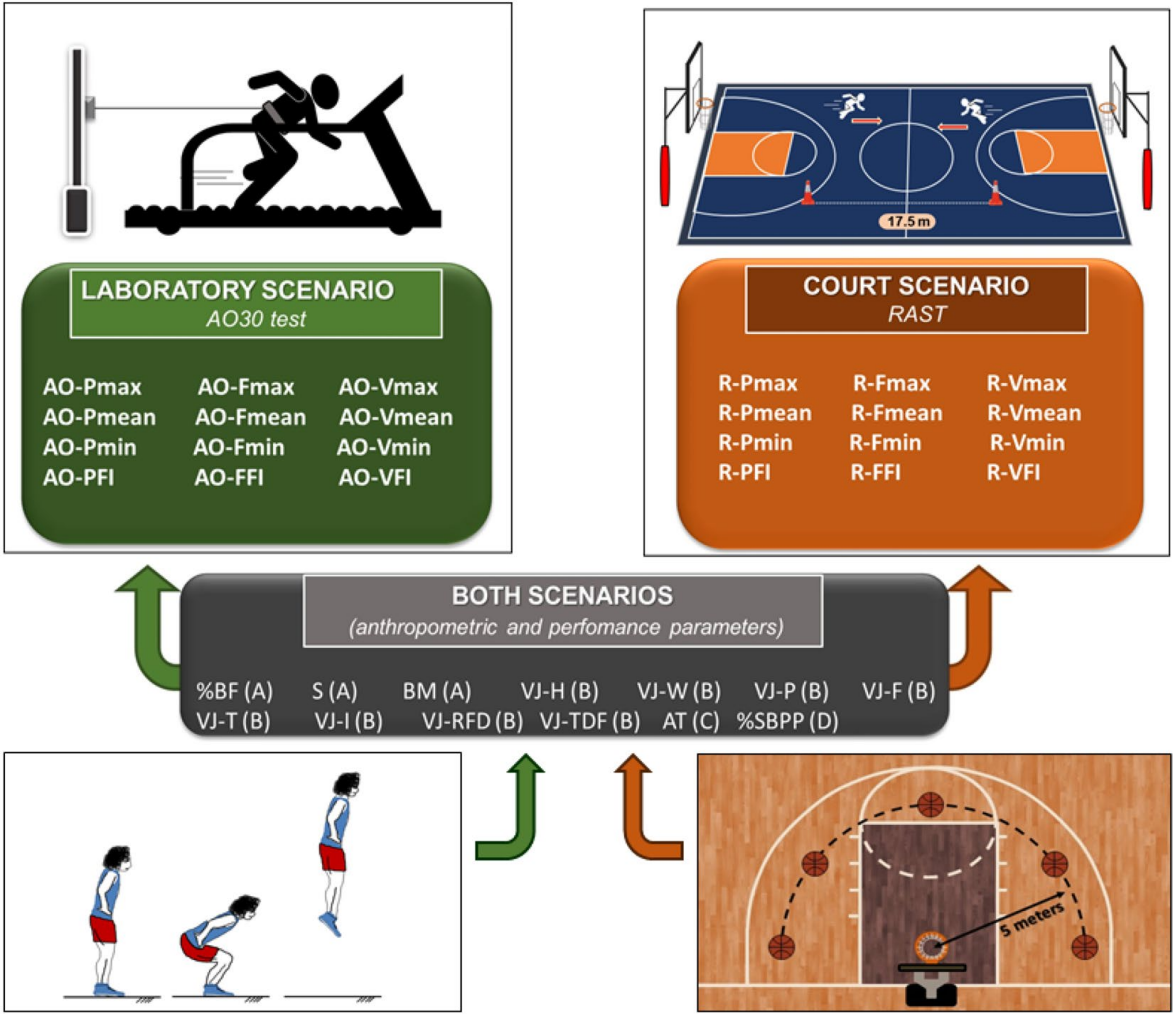
Specific parameters to Scenarios 1 (laboratory) and 2 (on-court), and the common parameters used in complex networks applied in both scenarios. The parameters at the same line (to Scenarios 1 and 2) are equivalents in mechanical terms. The common parameters include anthropometric characteristics (**A**) and countermovement jumps (**B**), anaerobic threshold (**C**) and technical performances (**D**). Legend - Scenario 1 (laboratory): AO-Pmax = All-out 30 s maximal power, AO-Pmean = All-out 30 s mean power, AO-Pmin = All-out 30 s minimum  power, AO-PFI = All-out 30 s power fatigue index, AO-Fmax = All-out 30 s maximal force, AO-Fmean = All-out 30 s mean force, AO-Fmin = All-out 30 s minimum force, AO-FFI = All-out 30 s force fatigue index, AO-Vmax = All-out 30 s maximal velocity, AO-Vmean = All-out 30 s mean velocity, AO-Vmin = All-out 30 s minimum  velocity, and AO-VFI = All-out 30 s velocity fatigue index. Scenario 2 (on-court): R-Pmax = RAST maximal power, R-Pmean = RAST mean power, R-Pmin = RAST minimum power, R-PF I = RAST power fatigue index, R-Fmax = RAST maximal force, R-Fmean = RAST mean force, R-Fmin = RAST  minimum force, R-FFI = RAST force fatigue index, R-Vmax = RAST maximal velocity, R-Vmean = RAST mean velocity, R-Vmin = RAST minimum  velocity, and R-VFI = RAST velocity fatigue index. Common parameters to both scenarios: %BF = percentage body fat, S = height, BM = body mass, VJ-H = vertical jump height, VJ-W = vertical jump work, VJ-P = vertical jump power, VJ-F = peak vertical jump force, VJ-T = vertical jump time, VJ-I = vertical jump impulse, VJ-RFD = peak rate of jump force development, VJ-TFD = time of jump force development, AT = anaerobic threshold, and %SBPP = success percentage of the shooting basketball performance protocol.

Finally, the third evaluation session was carried out on the basketball court and initiated with similar warm-up and recovery procedures. After that, participants performed the basketball technical test, consisting of the shooting basketball performance protocol (SBPP), with 10 two-point shots at five different places on the court. After 45 minutes, the athletes underwent the same warm-up and recovery when starting the running anaerobic sprint test (RAST). Next, and using the RAST as acidosis inducer, the anaerobic threshold (AT, aerobic capacity) determination was accomplished using the lactate minimum protocol, according to Camargo *et al*.^16^. So, the experimental design included two scenarios for complex networks analysis with analogous anaerobic parameters, one obtained from laboratory procedures and the other at the basketball court. Both scenarios also included the “common” parameters, including anthropometric characteristics, countermovement jumps, aerobic capacity and basketball technical shot performances. Figure 1 shows the common and scenario-specific parameters.

### Countermovement jumps

The athletes performed three maximal countermovement jumps (15 s among them) without aid to the arms on a force platform (signal capture of 1000 Hz). The force signals that the subject reached during the highest vertical jump (VJ-H) were chosen to determine other parameters using an algorithm in the MATLAB environment. Thus, in addition to the VJ-H, the vertical jump (VJ) variables were determined as work (VJ-W), power (VJ-P), force (VJ-F), time (VJ-T), impulse (VJ-I), rate of jump force development (VJ-RFD) and time of jump force development (VJ-TDF). All results from these parameters were included in the complex networks analysis in both the L and C scenarios (Fig. 1).

### All-out 30 s on non-motorized treadmill (NMT)

During the 30 s test, the subjects ran tethered to a horizontal force determination system by a calibrated sensor attached to the structure positioned behind the treadmill. Another set of force sensors (four load cells) were positioned at the base of the treadmill, aiming to obtain the vertical force component applied by the athletes throughout running. Thus, the force resultant was obtained by the vector sum of the vertical and horizontal sensor responses. All records were captured at 1000 Hz. The treadmill was also equipped with Hall-effect sensors to determine the linear displacement and thus velocity, and all signals were synchronized and conditioned by an extensometer source and integrator module (National Instruments). The records were captured by LabVIEW SignalExpress software, and the signals were analysed from an algorithm in the MATLAB environment. Power was obtained by the product of force and velocity data from the flat NMT ergometer. For this evaluation, upon arriving at the laboratory, the athletes were kept at rest for 10 minutes and then underwent the warm-up, running at 7 km/h for 5 minutes, and then kept at rest for 5 more minutes. After this, the athletes were tied by the waist to an inextensible cable (linked to the horizontal force sensor) and instructed to run as fast as possible for 30 s on the NMT. The participants were verbally encouraged to maintain the maximal speed throughout the all-out test. Data obtained from this analysis were the maximal, mean and minimum power, force and velocity measurements, as well as fatigue indexes (FIs) for each one of these mechanical parameters. The FI was obtained as the percentage of the difference from highest to lowest value divided by the highest result.

### Shooting basketball performance protocol percentage (%SBPP)

The SBPP technical performance test was arranged according to the two-point stationary test validated by Posjkic *et al*.^19^. SBPP consisted of 10 jump shots at five different positions on the basketball court (see Fig. 1, shooting positions), which were all five metres away from the centre of the rim of the basket. This protocol evaluated the shooting performance (percentage of success) of basketball players in a static condition, keeping the athlete at each position as shown in Fig. 1, and when the athlete would receive the ball from a partner under the basketball table, jumped and shot the ball toward the basket. The shots were accomplished after a 10 minutes warm-up freely shooting balls toward the basket at different distances. Only after this, the athletes were informed of the protocol and the placement of shots. Throughout the test, the athletes shot the balls continuously (two balls for each place) while changing the shooting position until the final shot (10^th^ ball). There was no time limit to complete the shots.

### Running anaerobic sprint test (RAST)

The RAST that was adapted consisted of six maximal 35 m running round trips, divided into two 17.5 m shuttle runs, and interspaced by 10 s of rest^16^. The times (t) obtained for each effort were recorded manually by an experienced appraiser using a digital stopwatch, positioned at the finish line, to determine the mean velocity (V = 35/t) of each sprint, as well as acceleration (a = V/t), force (F = body mass × a) and power (P = F × V). Like the laboratory all-out 30 s protocol, the athletes were verbally encouraged to maintain the maximal speed in each sprint. So, the mean, maximal and minimum values were determined to the same mechanical parameters as in the laboratory test. The fatigue index was determined for each V, F and P, by the percentage decrease from the maximal value to the minimum value. Blood samples (25 µL) were collected from the earlobe at rest and after 3, 5, and 7 minutes to determine the peak lactate concentrations ([LAC]) (YSI 2300 STAT Plus).

### Anaerobic threshold (AT)

To determinate the AT parameter, the lactate minimum protocol was applied. So, after 8 minutes of passive recovery from the RAST and the hyperlactatemia phase, an incremental intensity test was started on-court using metronome-controlled 20 m shuttle runs. The initial velocity was equivalent to 8 km/h, and 1 km/h was added every 3 minutes until exhaustion. Verbal instruction was given throughout the test with emphasis at the moment of the intensity increase, so that the velocity adjustment was attained. Blood samples (25 µL) were collected from the earlobe after each step of the incremental intensity test. Thus, at the end of each intensity in the incremental test, blood samples (25 µL) were collected to determine [LAC] (YSI 2300 STAT Plus). Blood lactate values were plotted against time from peak concentration, and the second order polynomial fit was determined, as well as exercise intensity when the derivative of this function was null^15^. This intensity can be considered the AT, which is the maximal running aerobic capacity.

### Complex networks analysis

Analysis by complex networks was performed using software (Gephi 0.9.2) after data processing by specific algorithm for this purpose in the MATLAB environment. The networks presented 25 nodes and, like a threshold as generally purposed on correlation networks construction^20^, their edges were obtained by the statistically significant “r” values (P < 0.05), from all results evaluated by Pearson correlation in each scenario, laboratory (L) and on-court (C), added to the common parameters in both (see below in statistical analysis). The notion of centrality originated from the analyses of social networks and is currently being used for metric interpretations of networks involving different actors. Thus, an undirected weighted graph G = (V, E, w), like used here, V represents the actors, E the edges that connect the actors, and w is the weight function^21^. In our case, V represents the nodes that involve, in both scenarios, absolutely similar parameters, including others that were integrated into the scenario networks in a common way (all nodes can be seen in Fig. 1).

In the present study, we used some centrality measures considered classic in complex networks models. A measure of centrality is the function c: G(n)→R^n^, where c*i* (*g*) is the centrality of node *i* in network *g*. The first measure of centrality presented in the study is the degree (d), representing the number of edges of node *i*, d*i* (*g*) that connects to the other nodes. Another measure of centrality used here, called betweenness, considers in an undirected weighted graph G = (V, E) being *n* and *m* the number of vertices (nodes) and edges, respectively. Thus, for a path *s*ЄV and *t*ЄV, *d*G (*s, t*) denotes the distance (*d*) between nodes *s* and *t*. Assuming that *d*G (*s, s*) = 0 for any *s*ЄV, *d*G (*s, t*) = *d*G (*t, s*) for any *s*, *t*ЄV, and that σ*st* = σ*ts* is the shortest path from sЄV and tЄV, where σ*ss* = 1 convention, and σ*st* (*v*) the shortest path number from *s* to *t* going through some possible *v*ЄV, then the betweenness centrality is the sum of the ratio σ*st*(*v*) by σ*st*, being *s* ≠ *v* ≠ *t*, all ЄV^22^. Another measure of centrality used in the present work is the eigenvector, which is an expansion of the “degree” metric. This considers the importance of the node connected to others in the network, in a prestigious representation in relation to its neighbourhood. It assigns, therefore, a value of the intensity of the connection between the nodes, evidencing the more critical ones among other vertices connected in a network. The higher the value is, the more prestige that node has over other prestigious in the graph^21^.

The fourth measure used was pagerank, the most recent algorithm compared to the others, which was developed by Google’s founders when students at Stanford University^21^ and is currently used as the primary tool on Google’s search pages. Pagerank is an analysis that seeks to identify the influence of a node through the importance of the connections it establishes. Thus, a high pagerank value assumes that a node acts critically when it is linked to others that perform essential functions in the studied network, assigning probability distributions to each node, so that its importance is not necessarily established by the number of node connections, but rather which other nodes are connected to it. Thus, a node can have high a pagerank score even though it is connected to few but especially important nodes. Therefore, it is a metric that highlights the best quality node in terms of information that the network can establish^21,23^.

In the present study, the construction of the graph applied the Fruchterman-Reingold^24^ layout in both scenarios. This option considered that this algorithm has a main objective to establish the most balanced distribution of nodes in the available space, trying to minimize the intersection of the edges and to establish uniformity and symmetry to the graph, making its analysis easier.

### Statistical analysis

Descriptive data are presented as the mean ± standard error of mean (SEM). Before statistical descriptive, product-moment correlation and complex networks analysis, the anthropometric, mechanical and performance results were submitted to the Kolmogorov-Smirnov test, showing P values that characterize data normality. So, the paired t-test was used to compare the corresponding assessment results of the mechanical parameters obtained from laboratory (AO30s) and on-court (RAST test). Also, product-moment correlation analysis (Pearson’s correlation) was performed among all the results of the specific parameters obtained from the laboratory and on-court added to the common parameters data in both. In all cases, the level of significance was P < 0.05.

## Results

As reported before, the AO30s and RAST protocols aimed to obtain mechanical parameters in running exercise emulating the maximal anaerobic effort as accomplished by the cycle ergometer proposed by Bar-Or^11^, which is also known as the Wingate test. Table 1 shows these results, with paired mean comparison (t-test) and correlations statistics (product-moment analysis). Our results found protocol dependence on data, since the mechanical results were different between evaluations in the laboratory and basketball court. On the other hand, like expected, the results of power and force (maximal, mean and minimum) were statistically highly correlated among the AO30s (laboratory) and RAST (on-court) protocols. However, there were non-significant correlations from the velocity parameters among the tests.

**Table 1:** Results of mechanical parameters obtained in the laboratory and basketball court from AO30s and RAST protocols applied in basketball players (N = 13).

Parameters	AO-30s (Laboratory)	RAST (On-court)	Paired t-test *P values*	Pearson’s r (*P values*)
**Power**				
Maximal power (W)	2497.9 ± 135.0	320.6 ± 22.4	*<0.01*	0.62 (*0.03*)
Mean power (W)	1894.0 ± 111.7	257.9 ± 13.4	*<0.01*	0.82 (<*0.01*)
Minimum power (W)	1387.9 ± 80.1	210.7 ± 12.8	*<0.01*	0.80 (<*0.01*)
Power fatigue index (%)	45.7 ± 1.1	18.1 ± 2.8	*<0.01*	0.06 (*0.83*)
**Force**				
Maximal force (N)	466.9 ± 20.9	59.0 ± 3.5	*<0.01*	0.67 (*0.01*)
Mean force (N)	409.6 ± 17.9	50.3 ± 2.3	*<0.01*	0.90 (<*0.01*)
Minimum force (N)	365.6 ± 16.5	44.7 ± 2.4	*<0.01*	0.76 (<*0.01*)
Force fatigue index (%)	23.1 ± 1.2	13.6 ± 2.8	*0.01*	0.17 (*0.57*)
**Velocity**				
Maximal velocity (m/s)	5.6 ± 0.1	5.4 ± 0.1	*0.12*	0.47 (*0.10*)
Mean velocity (m/s)	4.7 ± 0.1	5.0 ± 0.1	*0.02*	0.32 (*0.28*)
Minimum velocity (m/s)	3.6 ± 0.1	4.7 ± 0.1	*<0.01*	0.50 (*0.08*)
Velocity fatigue index (%)	35.9 ± 1.1	12.6 ± 1.6	*<0.01*	0.19 (0.53)

Results are mean ± SEM. Statistical significance P < 0.05.

**Figure 2:**
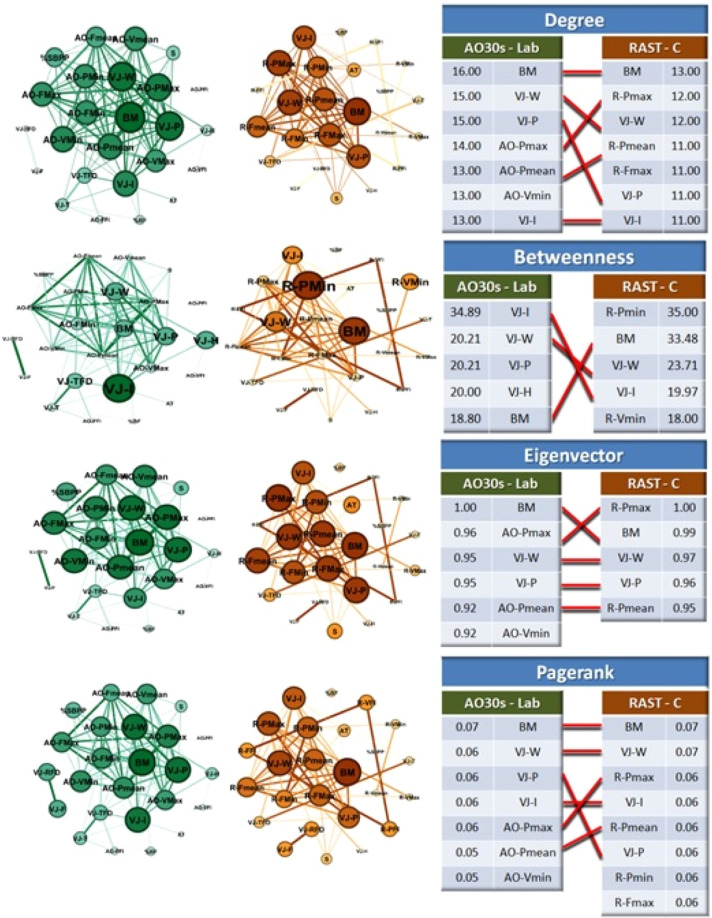
Graphs of centrality measurements of degree, betweenness, eigenvector and pagerank of two scenarios (laboratory-green and on-court-brown) involving mechanical and performances evaluations parameters of basketball players. At right side, tables showing the distribution values and the paired correspondent parameter linked by red line. Graph visualizations were done using Gephi software (version 0.9.2).

The results of centrality distributions are presented in Fig. 2. In the two scenarios, graphs of degree, betweenness, eigenvector and pagerank network are closed with tables showing the top five nodes that represent critical importance in each scenario based on metrics axioms. When the metrics values presented similar results for more than five nodes, the tables include all of them. Also, the tables include links throughout the similar parameters among the scenarios. From all parameters (nodes) analysed in each scenario, in 25 appearances of the total distributions used, there was a high resemblance of results in terms of parity and similarity between the scenarios.

For the laboratory results, in all the metrics used, BM, VJ-W and VJ-P appeared as top five in 100% of the analysed centrality distributions. Still in this scenario (L), the occurrence for AO-Pmax, AO-Pmean, AO-Vmin and VJ-I was 75% (three in four metrics), and 25% for VJ-H. In the case of the basketball court scenario (C), the parameters (nodes) BM and VJ-W appeared in all distributions (100%). In C (on-court), R-Pmax, R-Pmean, VJ-P and VJ-I appeared in three of four distributions (75%), while R-Fmax and R-Pmin appeared in 50% of the metrics used and R-Vmin in 25%. Also, among the scenarios (L and C), of the total of 25 nodes principal appearing in both, there was internal similarity (within each metric) in 80%, the most similar in terms of results being the degree and pagerank distributions (86% similarity). Also in this regard, the metric with the lowest similarity was betweenness, with 60% agreement.

**Figure 3:**
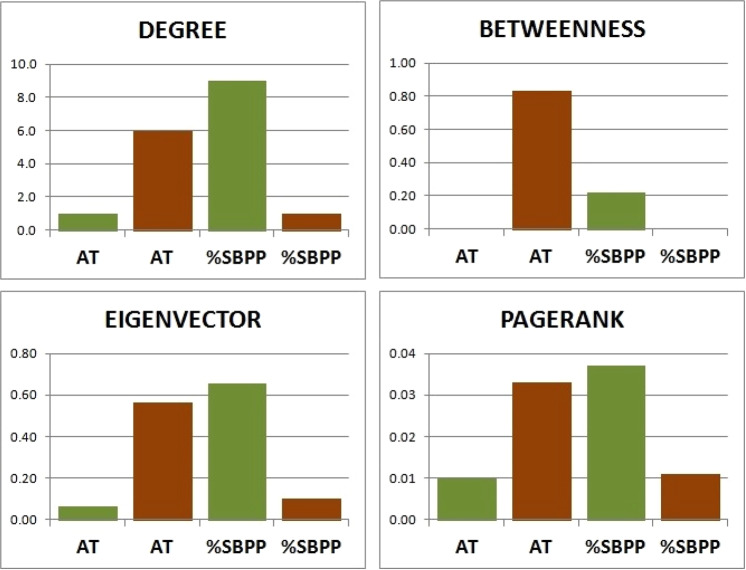
Centrality values to degree, betweenness, eigenvector and pagerank distributions for anaerobic threshold (AT) and shooting at basketball performance percentage (%SBPP) nodes in both scenarios (L in green, C in brown).

Figure 3 presents results obtained in the different centrality distributions for the AT and %SBPP nodes. The first one represents aerobic capacity, and the second, the specific technical performance of basketball. Although these nodes were not prominent in terms of centrality, their results were very different among the chosen metrics and seemed to have inverse levels between the two scenarios.

## Discussion

To the best of our knowledge, the present study is the first to relate physiological, mechanical and specific performance parameters in basketball using the complex networks model. Thus, this study focused efforts on comparing, by means of centrality metrics (degree, betweenness, eigenvector, and pagerank), tests that can successfully establish power, velocity, and force results in a sport assessment typically related to the anaerobic system. For each of these parameters, following for running what Bar-Or^11^ validated for the cycle ergometer, it is possible to determine maximal, mean, minimum values and fatigue indexes^13,14^. The first (maximal results) is related to anaerobic alactic metabolism, the second (mean results) to anaerobic lactic metabolism and the fatigue index is closely linked to acidosis tolerance. Therefore, the all-out 30 s test is an anaerobic evaluation accomplished in the laboratory and especially robust in its accuracy. The RAST protocol, corresponding test to the all-out 30 s test in the field, consists of six maximal 35 m runs, separated by 10 s intervals. For each of the sprints, it is possible to determine the values of the parameters of power, force and velocity, thus being able to establish the maximal, mean and minimum values and the respective fatigue indexes.

By submitting basketball athletes to both tests (AO30s and RAST), it was expected that the results of such mechanical parameters should be, at least, significantly correlated, since they are originally designed as corresponding assessments. In Table 1, it was possible to verify that the dimension of the values of the parameters of power and force were greater in the AO30s (laboratory) than in the RAST (on-court). These results corroborate with previous studies, even for applications of the RAST in its original form, characterizing a protocol-dependency previously reported even when efforts involve accelerations and decelerations compared to continuous efforts^15^. Despite this difference in a dimensional way, our experimental design depended on the presence of significant correlations between the laboratory and on-court results, without which the proposal would strongly lose the possibility of comparing the scenarios. In this sense, and according to our hypothesis, all the results for power and force were highly correlated.

In the case of velocity, except for the maximal results, the values relating to the Vmean and Vmin obtained on-court were higher than the corresponding ones established in the laboratory (6% and 23%, respectively). Previous studies have shown lower values in the maximum and mean velocities in running performed in NMT when compared to the field, with variation around 20%, in efforts involving high-intensity sprints^25–27^ and endurance running^28^. This response may be due to some factors such as the intrinsic resistance of the treadmill belt^25–27^, the shorter length and number of strides^25^, the friction between the track belt and the floor support structure during foot contact, as well as the inertia of the roller-belt system during strides and in the aerial phase of running^26,28^. These factors involving inertial elements probably justify the greater variation in the minimum velocity (23%, laboratory vs. court) observed in the present study. The authors also report that athletes with greater body mass have advantages running on the NMT since the inertial elements cause, relatively (per kg), a greater effect on lighter athletes^25–28^. This aspect may explain the prominent distribution of body mass in the four centrality metrics (Fig. 2) analysed in our study (in both scenarios), especially considering that body mass is one of the factors in determining force and power in the RAST.

Still regarding the velocity, our data also did not reveal significant correlations between the results in the AO30s and RAST protocols. Highton *et al*.^27^ investigated the 30 m performance times on the NMT and over ground, as well as splits of 10 m and 20 m. Of the 12 velocity parameters obtained, the authors observed significant correlations between the laboratory and the field in only half, with the correlation coefficients considered moderate in five (r = 0.58–0.67) and high for one of the parameters (time of 30 m, r = 0.80). On the other hand, Stevens *et al*.^28^ reported strong correlations (r = 0.82) between times obtained in endurance running (5000 m) from the NMT and over ground athletics track.

In our case, the lack of significant correlations among velocities for the RAST and AO30s tests may be due to the development of the running velocity to be quite different between the tests. For the RAST, we used the adapted protocol for the distance of 35 m (i.e., 2 × 17.5 m), featuring six sprints (10 s rest among them), including phases of acceleration (concentric force), deceleration (eccentric force), 180° change of direction (isometric contraction) and new acceleration, characteristic of the shuttle run^29^. Despite this, the RAST did not seem to generate a change in net energy cost when considering 17.5 m efforts, based on the report by Zamparo *et al*.^5^ involving young basketball athletes in shuttle runs with change of direction (180°). Considering that, in running on the NMT, there are no changes of direction, it is possible that this aspect, fundamentally methodological, is a strong candidate to explain the non-significant correlations for velocities between the AO30s and RAST protocols. This point needs clarification and should be further investigated in future studies.

Regarding the results obtained from the centrality analysis in complex networks, the 25 specific and common scenario parameters (nodes), which resulted from Pearson correlations (with the criteria of being statistically significant) returned 104 edges for scenario 1 (AO30s - laboratory) and 74 edges for scenario 2 (RAST - on-court). It was possible to verify a huge similarity between the scenarios in the different centrality metrics. In this sense, BM and VJ-W were present as the most important nodes in all the metrics used (degree, betweenness, eigenvector and pagerank). In a complex networks approach involving aerobic and anaerobic efforts in sprint athletes, Pereira *et al*.^30^ found a result of greater importance for BM in the measurement of betweenness centrality, and this parameter also ranked among the six most important in the other centrality metrics, leading the authors to highlight the relevance of anthropometric parameters in sports scenarios when analysed using this model. Delextrat and Cohen^3^ conducted a study involving basketball in an experimental design very similar to that of the present investigation, where they also related laboratory and field results, including vertical jump, the Wingate anaerobic test (on the cycle ergometer), 20 m sprint, agility test, suicide sprint, isokinetic (knee extensors) and one maximal repetition (1-RM, bench press). These authors verified that, for the current rules of basketball, the VJ figured as one of the main performances for basketball, as well as others of anaerobic power, determined by different tests from the present work. Gallová *et al*.^31^ compared the performances of Slovak and Lithuanian adolescent basketball players using the CMV and 20 m sprints and assumed these as tests which relate well to the game demands. More consistently and corroborating this view, our results highlight these performances in a network analysis.

In the specific case of basketball, besides the values of BM and VJ-W, it was possible to find in both scenarios that the other vertical jump nodes (VJ-I and VJ-P) for the CMV test also presented high centralities for the metrics involved in the analysis. Thus, all vertical jump nodes were among the main ones for degree and pagerank metrics in both scenarios, and for betweenness, the vertices VJ-W, VJ-P and VJ-I appear as main ones in the AO30s scenario, with corresponding VJ-W and VJ-I appearing in the RAST scenario. In the case of the eigenvector measure, VJ-W and VJ-P appear in the same prestigious positions in both scenarios. These results showed that the complex networks analysis was sensitive to these parameters and so important for basketball in the laboratory and field tests. In a review study, Drinkwater *et al*.^4^ reported that, in a basketball match, an athlete performs around 50 jumps, and this skill should be understood as fundamental in match performance^31,32^, along with other high power activities, especially 10–20 m sprints^4,33^.

In this sense, our results add that the test models used revealed, besides the vertical jumps, the presence of the maximal and medium powers as parameters of high and intense connection, as well as of prestige of these parameters regarding the others analysed in the present study, as shown in both scenarios for the degree, eigenvector, and pagerank measures. Since these vertices represent alactic and lactic anaerobic characteristics, respectively, the association with the specific profiles of the sport, played in 28 m courts, also reveals the extreme quality of the tests applied in both the laboratory and basketball court. It has been discussed in the literature which are the best optimal sprint distances to test athletes of this sport, being found to be distances of 10 m to 27 m^4^. Since it is suggested that acceleration, deceleration and change of direction are key components of the specific demands imposed on the basketball game^34^, power should be considered as a relevant parameter for the sport. Therefore, we can assume that both the all-out 30 s test and RAST adapted to the court dimensions generate extremely adequate results in terms of evaluation. The on-court test is especially interesting for this purpose for it includes, as already reported, sprints from repeated acceleration stimuli.

According to this definition, regardless of the distances suggested for the tests, the protocols from different scenarios were well associated regarding centrality metrics for these maximal and mean power parameters (except for betweenness), suggesting that the on-court test reveals results that, in an integrative way, are compatible to the technological tests performed in the laboratory. This strongly indicates that the simpler on-court protocol gives, at least considering a network scenarios perspective, the same results as those obtained in the laboratory. In terms of practical application, the field protocol is highly attractive, as it meets demands of interest that are widely usable and robust, such as a laboratory evaluation for these mechanical and metabolic profiles.

Figure 3 shows the results of the four metrics for the AT (aerobic capacity) and %SBPP (technical performance) nodes. This figure was especially presented to show that, although these parameters have no prominent representation in the analysis, the complex networks model was sensitive in showing differences between scenarios. The AO30s, as already reported, is a predominantly anaerobic exercise protocol, as is the execution of the maximal vertical jumps and basketball shots. Not surprisingly, the participation of AT in this context was extremely small in the laboratory setting, but was much higher when participating in the basketball court network. In contrast, %SBPP had a higher representation in the laboratory scenario than in the on-court scenario. Again, it is important to revisit the different protocols studied in order to discuss these data, which were provocatively included here. In the RAST, unlike the AO30s, athletes perform maximum 35 m (2 × 17.5 m in adapted form) back-and-forth runs with breaks of 10 s. Such intervals allow greater oxidative activity in this test as compared to the laboratory test. It is amazing to see that, in the complex networks model, this metabolic detail was sensitive enough in the four different centrality metrics. In contrast, since basketball shooting involves alactic anaerobic predominance, the %SBPP was much higher in the laboratory setting than in the on-court scenario for all centrality measures. Viewed this way, our study also denotes the incredible sensitivity of the complex networks model in a task accomplishment analysis, which would hardly be possible in a conventional statistical analysis. Taken together, these results allow us to establish a broad perspective on the application of the metrics used in the sports scenario, in particular by showing that the possibilities of interpretation of the obtained values are extremely consistent with the physiological representations pertinent to the proposed evaluation protocols.

In summary, the results (maximal, mean, minimum values and fatigue index) for force, velocity and power obtained in the laboratory and on-court tests included other anthropometric and performance parameters, such as aerobic capacity, vertical jump, and specific technical (successful shot-to-basket), showed, in an incredible way, the correspondence of centrality metrics between the scenarios. The parameters BM, VJ-W, VJ-I, Pmax and Pmean were the most prominent among the applied complex networks measurements (degree, betweenness, eigenvector and pagerank). Moreover, from these analyses, we can conclude that, at least in our experimental design, the RAST adapted for the basketball court is a protocol that fulfils its role very well, which for its simplicity on implementation should be attractively used by basketball teams. Thus, in practical terms, basketball being a modality that involves high-intensity intermittent exercise in tasks that involve a high frequency of changes in direction, the RAST is confirmed as an important assessment tool for this sport, especially for young athletes who need muscle preparation capable of withstanding lower limb rotational demands, high anaerobic power and tolerance to acidosis.
